# Poly (Lactic Acid) membrane and Sedum dendroideum extract favors the repair of burns in rats^1^

**DOI:** 10.1590/s0102-865020200030000002

**Published:** 2020-05-11

**Authors:** Juliane Peliçari Binotto, Larissa Giorgetti Mendes, Fernanda Oliveira de Gaspari Gaspi, Marcelo Augusto Marreto Esquisatto, Thiago Antonio Moretti de Andrade, Fernanda Aparecida Sampaio Mendonça, Gláucia Maria Tech Santos

**Affiliations:** I Fellow Master degree, Graduate Program in Biomedical Sciences, Centro Universitário, Fundação Hermínio Ometto (FHO), Araras-SP, Brazil. Acquisition and interpretation of data, technical procedures, histopathological examinations, statistics analysis, manuscript preparation.; II Master, Department of Materials Engineering and Bioprocess, School of Chemical Engineering, Universidade Estadual de Campinas (UNICAMP), Brazil. Membrane preparation, interpretation of data, manuscript preparation.; III PhD, Centro Universitário, FHO, Araras-SP, Brazil. Preparation of herbal extract, interpretation of data, manuscript preparation.; IV PhD, Graduate Program in Biomedical Sciences, Centro Universitário, FHO, Araras-SP, Brazil. Histopathological examinations, statistics analysis; manuscript writing, critical revision, final approval.; V PhD, Graduate Program in Biomedical Sciences, Centro Universitário, FHO, Araras-SP, Brazil. Histopathological examinations, interpretation of data, critical revision.; VI PhD, Graduate Program in Biomedical Sciences, Centro Universitário, FHO, Araras-SP, Brazil. Analysis and interpretation of data, manuscript writing, final approval.; VII PhD, Graduate Program in Biomedical Sciences, Centro Universitário, FHO, Araras-SP, Brazil. Conception and design of the study, manuscript preparation, critical revision.

**Keywords:** Wound Healing, Burns, Phytotherapy, Skin, Rats

## Abstract

**Purpose:**

To evaluate the healing potential of the electrospinning membranes of Poly (Lactic Acid) (PLA) associated with *Sedum dendroideum* extract in burn injuries in rats.

**Methods:**

Seventy-five rats were submitted to burn injury on their back skin: (**C**) untreated; (**F**) with daily topical application of *S. dendroideum extract*; (**M**) with electrospinning membranes of PLA; (**MF10**) with electrospinning membranes of PLA with 10% *S. dendroideum extract*; (**MF25**) with electrospinning membranes of PLA with 25% *S. dendroideum extract*. Tissue samples were taken after 2, 6 and 14 days of the burn injury and were subjected to histomorfometric analysis of quantification of fibroblasts, collagen fibers, blood vessels, and inflammatory infiltrate

**Results:**

The histomorphometric analysis showed an increase in the number of fibroblasts, collagen fibers and blood vessels in the burns treated with membranes of PLA, associated or not with the 10% and 25% extract. The extract of *S. dendroideum* promoted the increase of collagen fibers.

**Conclusion:**

The electrospinning PLA membrane, isolated or associated with the *S. dendrodeum* extract, favored the healing of burn injuries in this experimental model, with an increase of fibroblasts, collagen fibers, and blood vessels. *S. dendroideum* isolated only stimulated the collagenesis.

## Introduction

Burns of II-IIIa degree makes the majority of thermal injuries when treatment is mainly carried out by conservative methods. The problem of the covering of large burned surfaces is still a challenge^1^ ^,^ ^2^. The treatment of burn includes removal of dead tissues (debridement), dressing of the wound, fluid recovery, antibiotics administration, skin grafting (autografts, allografts, and xenografts), cultured dermal substitute and transplantation of keratinocytes, besides dressings like Silver Sulfadiazine and specific herbals^3^. A wide variety of preparations and remedies of non-organic, organic, biogenic, and phytogenic origin have been devised and used in the topical treatment of burns^4^.

More recently, tissue engineering has been indicated in the treatment of burns, suggesting modern biopolymer-skin substitutes to create favorable conditions for wound epithelialization, eliminating the discomfort caused by pain at dressing changes, and preventing the effect of pathogenic microflora. Furthermore, the wound heals faster when it is stored in a humid environment, because the exudate is a key component in all phases of wound healing. It is responsible for delivering nutrients to the wound and creating favorable conditions for migration and mitosis of epithelial cells, the proliferation of granulation tissue and fibroblast, migration of leukocytes, and it also enhances local immunity and wound cleansing, keeping endogenous bioelectric fields that affect the movement of cell^5^ ^,^ ^6^.

Electrospinning technology for non-fibrous membrane manufacturing has studied new nanostructured materials and their applications. Among them, a large part was intended for biomedical applications, such as tissue regeneration substrate, immobilized catalyst enzymes, artificial dressings, and blood vessels^7^. The ease of producing materials in the nanometer scale, size scale of biological materials, arouses great interest in this technique for application of membranes obtained in the medical field, such as in the covering of injuries and the controlled release of drugs^8^. Biopolymers are considered ideal material due to biocompatibility, biodegradability and easy applicability^2^ and Poly (Lactic Acid) (PLA) stands out for its low production cost, low or no toxicity and high mechanical performance^9^. In controlled release systems, nanofibers are considered as a potential drug carrier because they can be placed on surgical injuries or encapsulated for release into the digestive system. In periodontal disease, the first controlled drug release test was performed using Poly (Lactic Acid) (PLA), Poly (Vinyl Ethylene-Co-Acetate) (PEVA) and PLA/PEVA (50:50) nanofibers incorporated with the hydrochloride of tetracycline^10^. Electrospinning PLA nanofibers incorporated with turmeric (*Curcuma longa*) showed good results related to hydrophilicity, drug absorption, improving cell viability, adhesion, and proliferation, suggesting that they have healing potential for wound healing applications^11^. Electrospinning PLA scaffold and resveratrol promoted repair of cartilage injury in animal models^12^ and electrospinning PLA dressings also induced improvement in the healing process of mucosal defects in humans^13^. The development of functionally integrated multifunctional dressings for the treatment of burn wounds that can control infections and promote tissue reconstitution is a major challenge today. Making electrospinning PLA nanofibers is a promising strategy with excellent results^14^.

Herbal or medicinal plants can be used in controlled release systems through scaffolds. The therapeutic use of medicinal plants is very promising because it is inexpensive when compared to synthetic drugs^15^. *Sedum dendroideum* DC, popularly known as a balm in Brazil, is used as an ornamental plant and also in folk medicine for gastric ulcers, general inflammatory processes and also in tissue healing because it presents antinociceptive and anti-inflammatory activities demonstrating its ethnopharmacological value^16^ ^–^ ^18^.

Electrospinning PLA membranes used as scaffolds for release of *S. dendrodeum* extract promoted satisfactory responses in the healing of excision skin lesions^19^. However, it is important to evaluate the effectiveness of the electrospinning membrane in other models of experimental injuries, for example burns. Electrospun nanofibrous of chitosan and polyethylene oxide was proposed for burn dressing because it reduces the time of tissue repair and provides faster rehabilitation of patients^20^.

Therefore, this study aimed to evaluate the healing potential of the electrospinning membranes of Poly (Lactic Acid) (PLA) associated with *Sedum dendroideum extract* in second-degree burn injuries to the back skin of rats.

## Methods

### Plant material

*Sedum dendroideum* leaves were collected, in the month of February (summer), at the medicinal plant garden of Centro Universitário, Fundação Hermínio Ometto (FHO). The species was identified and a voucher was deposited at the herbarium of the Department of Biological Sciences, ESALQ/USP, under the identification number 115687. The registration in SISGEN is A270150.

### Extract preparation

After harvesting, the leaves were selected, cleaned, cut and grounded in a stainless-steel blender and then submitted to the hexane maceration extraction process at room temperature. After vacuum filtration, the filtrate was concentrated on a rotary evaporator to obtain the hexane extract which was incorporated into the PLA membrane. For the injury treatment only with *S. dendroideum* hexane extract was dissolved in 1:1 saline solution.

### Membrane preparation

The membranes were made at the Department of Material Engineering and Bioprocesses - UNICAMP, using electrospinning equipment composed of high voltage source (Testtech), syringe, 9KD-100 syringe pump, KD (Scientific) and a grounded copper collector. PLA solutions (19450 g/mol) in Dichloromethane (DCM) (Synth - Diadema, Brazil) were prepared as a solvent with a concentration of 7.5% w/v and at a solution flow rate of 6 mL/h.

### Preparation of membranes with incorporation of S. dendroideum extract

To obtain the membranes incorporated with the *S. dendroideum* extract, the solutions containing 7.5% w/v of PLA in DCM were added to the extract concentrations of 10% and 25% v/v. After stirring the solution, the membranes were made by the electrospinning process described above. The membranes were then cut in a 20 mm square shape and sterilized in ethylene oxide by ACECIL, and only after 72 hours were used in the animals. The choice of extract concentrations (10% and 25%) was determined by a result previously reported in the literature^19^.

### Animals

All surgical and experimental procedures used in this study were conducted in accordance with the experimental requirements and biodiversity rights of the National Institute of Health for the Care and Use of Laboratory Animals (NIH Publication 80-23, revised 1996). The studies were conducted according to the rules established by the Arouca Law and approved by the Animal Use Ethics Commission (CEUA) Fundação Hermínio Ometto, Centro Universitário (number 008/2012).

A total of 75 male Wistar rats (*Rattus novergicus albinus*) were obtained from the Animal Experimentation Center Fundação Hermínio Ometto, Centro Universitário; they were approximately 90 days old and had an average weight of 250 g. The animals were housed in individual polycarbonate cages and kept in a controlled environment with constant temperature (23±2°C) and humidity (55%), under a 12:12 hours light/dark cycle with free access to commercial standard feed and potable water.

### Burn Injury and experimental design

Dorsal region trichotomy was performed in all animals after anesthesia by intraperitoneal administration of Xylazine Hydrochloride (25 mg/kg) and Ketamine Hydrochloride (75 mg/kg). Subsequently, the burns were produced on the back skin of all animals by applying an aluminum metal plate (2 cm in diameter), adapted to an electrical device that maintains a constant temperature of 120° C. To establish the same burn pattern, a graduated support rod was used to support the aluminum plate with the same pressure on the animals’ skin. This plate was placed on the animal's back skin for 20 seconds to produce the second-degree burn^21^. The animals were then placed in individual cages and given Dipyrone Sodium (10 mg/Kg/12/12 hours orally) for 3 days.

The animals were randomly divided into five groups (n=15): (C) untreated control animals; (F) animals treated with topical application (1 mL/day) of the *S. dendroideum* extract dissolved in saline solution using a 1:1 proportion; (M) animals treated with electrospinning membranes of PLA; (MF10) animals treated with electrospinning membranes of PLA with 10% of *S. dendroideum* extract incorporated in the fibers; (MF25) animals treated with electrospinning membranes of PLA with 25% *S. dendroideum* extract incorporated in the fibers.

The treatments were started immediately after the experimental injury, at the same time and by the same researcher. *S. dendroideum* extract was applied daily with the aid of a Pasteur pipette dropping 1.0 mL over the injuries and, for this, the animals were only immobilized. Injuries using only the extract were not occluded, but those that received the membrane, associated or not with the extract, were occluded with sterile gauze. The burn area was not open in this experimental model and did not occur contamination during the study. This is justified by the excellent sanitary conditions of the animal facilities. The burn bed was not debrided before the application of the respective treatment and the crust was not removed but fell off by itself during the follow-up. The animals did not receive antibiotics so that there was no interference in the healing process of the burn that was the focus of the study.

After 2, 6 and 14 days of treatment, five animals from each group were euthanized by deepening of anesthetic (the same conditions above) associated with a cervical dislocation, and samples of the injuries were collected for histomorphometric analysis. For this, an area of 30 mm in diameter was delimited in the center of the injury to obtain standardized samples. After removal, the tissue fragments were immersed in a fixative solution containing 10% formaldehyde in Millonig pH 7.4 buffer for 24 hours at room temperature.

### Histomorphometric analysis

The fixed samples were washed in buffer and subjected to standard Paraplast (Histosec^®^-Merck) soaking procedures. Each block obtained from the samples was submitted to microtomy and 5 µm thick longitudinal sections were treated with the following techniques: Masson's trichrome (TM) (quantification of collagen fiber content in the repair area - percentage of total area); Toluidine Blue - AT (measurement of the number of fibroblasts and blood vessels); Dominici (quantification of inflammatory infiltrate) at 400x magnification, analyzed by Leica DM2000 Photomicroscope. Collagen fiber organization and maturation were evaluated by quantifying area of birefringence concerning the total area by the Picrosirius-hematoxylin method under polarized light^22^. For this study, three samples were chosen from each of the five sections obtained from the middle region of the tissue from the five animals in each experimental group (n=15 images/animal). To measure each of the parameters in the repair area, the Sigma Scan Pro 5.0 ™ program was used.

### Statistical analysis

The results obtained in the morphometric analysis were compared by the Two-way ANOVA test and Bonferroni post-test and performed using GraphPadPrism^®^ 6.0 software version with the comparison of all groups in each period with a pre-established significance level of 5% (p < 0.05).

## Results

**Figure 1:**
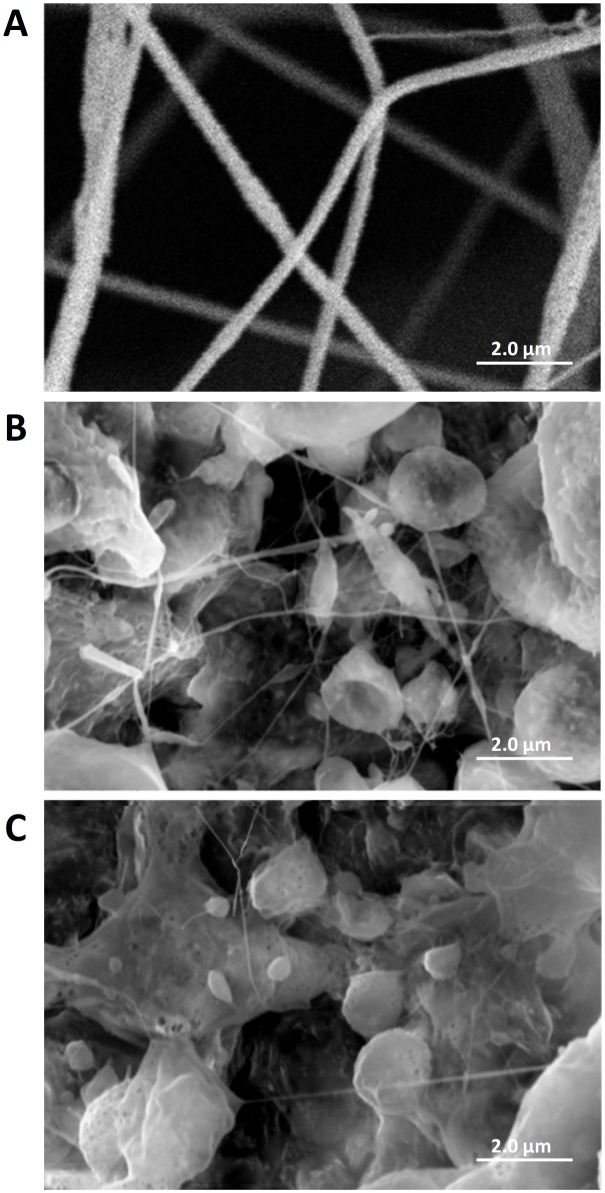
7.5% pure PLA membrane **(A)**; 7.5% PLA membrane with 10% *S. dendroideum* extract **(B)**; 7.5% PLA membrane with 25% *S. dendroideum* extract **(C)**; Images with x10.000 magnification.

The membranes obtained for this study did not present the expected morphology for the electrospinng technique, as can be observed in the SEM images (Fig. 1). The addition of *S. dendroideum* extract modified the properties of the solution, causing the formation of beads interconnected by nanofibers, in the membrane structure. However, the whole structure obtained showed a high degree of porosity, which is an excellent feature for use as a scaffold, since there is an increase of the contact surface for cell growth in the healing process.

**Figure 2:**
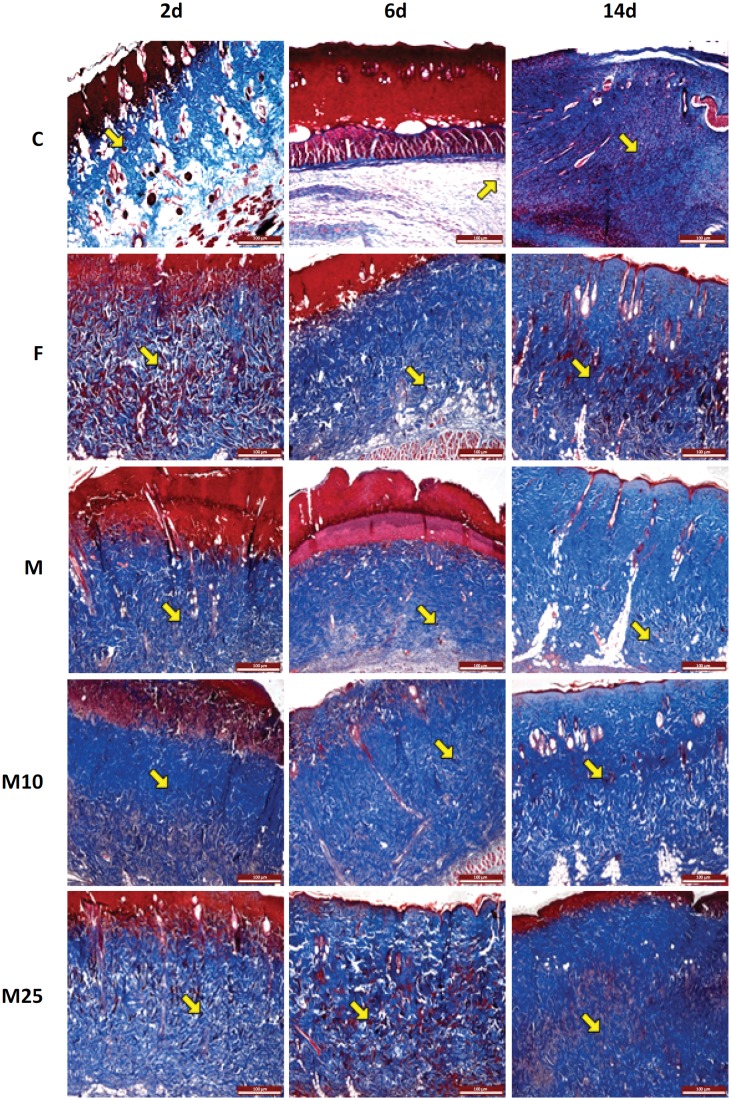
Cross sections of the second-degree burn injury area to the back skin of rats of the experimental groups: **(C)** untreated; **(F)** with daily topical application of *S. dendroideum* extract; **(M)** with electrospinning membranes of PLA; **(MF10)** with electrospinning membranes of PLA with 10% *S. dendroideum* extract; **(MF25)** with electrospinning membranes of PLA with 25% *S. dendroideum* extract. Tissue samples were taken after 2 (2d), 6 (6d) and 14 (14d) days of the burn injury. The sections were stained with Masson's trichrome. → – Inflammatory infiltrate. Blue Coloring - Collagen fibers. Bar = 500µm.

The histomorphometric data are gathered in Table 1 and illustrated in Figure 2.

**Table 1:** Morphometric parameters evaluated in the second-degree burn injury area to the back skin of rats of the experimental groups. (**C**) untreated; (**F**) with daily topical application of *S. dendroideum* extract; (**M**) with electrospinning membranes of PLA; (**MF10**) with electrospinning membranes of PLA with 10% *S. dendroideum* extract; (**MF25**) with electrospinning membranes of PLA with 25% *S. dendroideum* extract. Tissue samples were taken after 2 (**2d**), 6 (**6d**) and 14 (**14d**) days of the burn injury.

Times	2d					6d					14d				
Parameters/Groups	C	F	M	MF10	MF25	C	F	M	MF10	MF25	C	F	M	MF10	MF25
Inflammatory infiltrate (n/10^4^ mm^2^)	32±2	31±3	37±4	38±4* a	37±4	27±3	28±3	31±3	34±3* b	32±3	24±3	28±4	30±3	32±3* c	31±2
Number of fibroblasts (n/10^4^ mm^2^)	15±3	14±2	15±3	15±2	14±3	25±4	27±3	32±3	33±3* d	31±3	28±4	31±3	40±3	42±3* e	41±3
Number blood vessels (n/10^4^ mm^2^)	1.5±0.3	1.4±0.2	1.5±0.4	1.5±0.2	1.4±0.2	2.5±0.4	2.7±0.3	3.1±0.3	3.3±0.3* f	3.3±0.2	2.8±0.4	3.1±0.3	4.3±0.3* g	4.2±0.3	4.1±0.3
Birefringence collagen fiber (% of area)	20±8	24±7	27±7	29±7	28±7	39±11	41±8	42±8	40±8	39±8	53±9	68±6	78±7	80±8* h	79±10

Values were compared between experimental groups and periods using ANOVA, Tukey post-test at 5% significance level.

(*) Significant difference:

a p = 0.04 (2d);

b p = 0.048 (6d) and

c p = 0.042 (14d) - similar values between groups M, MF10 and MF25/C and F;

d p = 0.042 (6d);

e p = 0.041 (14d) - similar values between groups M, MF10 and MF25/C and F;

f p = 0.047 (6d);

g p = 0.043 (14d) - similar values between groups M, MF10 and MF25/C and F;

h p = 0.045 (14d) - similar values between groups F, M, MF10 and MF25.

The quantification of the inflammatory infiltrate presented higher values in the injuries of M, MF10, and MF25 in all experimental periods. The number of fibroblasts showed an increase in the injury samples of the groups that used PLA membrane associated or not with *S. dendroideum* on the 6th and 14th day after the injury. The same was observed regarding the percentage of birefringent collagen fibers only in the last experimental period including also the group treated with *S. dendroideum* only. Blood vessel quantification showed an increase in the 6th and 14th days, in groups M, MF10 and MF25 compared to groups C and F.

## Discussion

Given the difficulties in establishing a noninvasive therapy for the treatment of burns, new techniques are sought to promote rapid and effective occlusion for this type of injury, especially if the occlusives are healing^1^. Different polymers capable of interacting with cells have been developed to promote tissue growth and differentiation^8^. Among these the Poly (Lactic Acid) (PLA) stands out for promoting prolonged action in the tissue^23^.

PLA has good biocompatibility and degradation and can be degraded into carbon dioxide and water *in vivo*, which is widely used in the preparation of tissue engineering scaffolds and drug carriers, as also for wound healing through the high porosity that could facilitate nutrient and wastes exchange^24^. Biodegradable, biocompatible PLA nanofibers, with their morphological similarities to the extracellular matrix of the skin, have great potential to be used as skin substitutes and wound dressings^25^ ^,^ ^26^.

The characterization of PLA membranes obtained by electrospinning was performed by Santos *et al.* ^19^ who observed the formation of porous structures and the presence of beads along the fibers that enable the use of reservoirs for active release. The authors point out that PLA membranes incorporated with *S. dendroideum* extract (10 and 25%) do not have the same morphology as those obtained without the extract and described as composed of particles and beads connected with nanofibers, which lead to a porous structure. Kim *et al*.^27^ also obtained changes in the structure of electrospun membranes by incorporating antibiotic (Metoxin) in PLAG/DMF solutions, and could see the influence of the properties of the solutions used in membrane morphology. Despite this, the membranes showed a satisfactory result in Santos *et al*.^19^, who previously tested *S. dendroideum* incorporated PLA membranes in the healing of excisional skin lesions in Wistar rats.

In this study, injuries treated with the PLA membrane showed a positive response to fibroplasia. The increase in fibroblasts is important in injury regeneration, collagen production, elastin, glycosaminoglycans leading to tissue integrity^28^ ^,^ ^29^. Santos *et al.* ^19^ also obtained similar results regarding quantification, organization and maturation of collagen fibers, and number of fibroblasts in excision injuries treated with PLA membranes incorporated or not to *S. dendroideum* extract. It has also been found that PLA Scaffolds promote fibroblast proliferation^30^ and that nanoparticles accelerate wound healing by promoting fibroblast migration and collagen deposition^31^.

Increased collagen fiber content was also observed by Nitz *et al.* ^32^ in skin injuries of Wistar rats treated with herbal medicines (*Coronopu didymus* and *Calendula officinali*) that present flavonoids in their composition. Excision injuries also found an increase in the number of collagen fibers in the groups treated with the flavonoid fraction of *Sphaeranthus amaranthoides* ^33^. These observations may explain the data obtained in this study with the increase in collagen in samples treated only with topical application of *S. dendroideum*, which also has flavonoids in its composition^16^. Other herbal medicines promote increased collagen in skin and tendon healing^34^ ^–^ ^37^ which represents one of the most important factors for the tecidual repair after the lesion^38^.

The deposition of collagen fibers is favored by fibroblast proliferation, decreased inflammatory infiltrate, accompanied by the appearance of new blood vessels. In a continuous process, the wound bed is gradually replaced by the scar fibrous matrix resulting from the ordered deposition of the new collagen fibers. The latter are progressively remodeled by the action of matrix metalloproteinases until they reach the ideal maturation stage, restoring the functional aspects of the pre-injury dermis^39^. In this work, the structural aspects related to the collagen fibers deposited in the repair region indicated differences between the treatments and the control group. These data indicate that the therapies seem to affect the organization of the fibers in the tissue since the samples from the animals treated on the 14th day had a higher area occupied by birefringent and, consequently, more compact collagen fibers. These data indicate that the remodeling of the fibrous matrix occurred in a differentiated dynamics in the treated groups. This phenomenon can be explained by the possible effect of the therapies during the repair on the balance of electric charges in the extracellular environment, which seems to delay the compression of the collagen fibers during the repair phase about the control group. These data have the support from the work of Chao *et al.* ^39^, Lee *et al.* ^40^, and Xu *et al.* ^41^ who demonstrated how the electrical potential difference and laser irradiation causes physical-chemical stimuli in the tissue thus altering the tissue response regarding the synthesis, deposition, and organization of collagen fibers.

Data for blood vessel quantification, which showed a gradual increase in injuries treated with PLA membranes incorporated or not in the extract, corroborate with the results of Santos *et al.* ^19^ for excision injuries where the same treatment was used. These findings reinforce that the presence of the PLA membrane in contact with the injury promotes beneficial effects on the process of repair of burn and excision injuries, as a consequence of angiogenesis stimulation. Besides, Perez-Amodio *et al*.^42^ noted that the combination of Poly (Lactic Acid) (PLA) and Calcium Phosphate glass nanoparticles promoted angiogenesis, collagen deposition in chronic wounds in diabetic mice. PLGA (Poly-D, L-Lactide-Co-Glycolide) also promoted angiogenesis and accelerated closure of excision skin wounds in different mouse strains^43^.

Regarding the inflammatory infiltrate, it was observed that the use of membranes increased the beginning of the repair process. This result is supported by data from Rezende *et al.* ^23^ who studied the action on tissue repair of Poly (Lactic Acid-Glycolic Acid) membranes *in vitro* and *in vivo* and observed that these intensified the inflammatory phase of the process. The intense inflammatory response observed in our study detected until the last experimental period, is probably due to the presence of the PLA membrane. The same fact was observed by Santos *et al.* ^19^ in excision injuries submitted to the same treatment. The presence of these scaffolds *in vivo* probably stimulates the release of proinflammatory cytokines that promote the migration of inflammatory cells to the site^44^.

The incorporation of hexane extract of phytotherapic in the membrane in different percentages (10 and 25%) did not show different results in relation to the application of the membrane without the incorporation of the extract, which leads to the conclusion that the observed results are due to presence of the electrospinning membrane and not the extract.

## Conclusions

The PLA electrospinning membrane, isolated or associated with *S. dendroideum* extract, promoted angiogenesis, fibroplasias, and collagenesis in the process of tissue repair of second-degree burns on the back skin of rats. In turn, *S. dendroideum* isolated only stimulated the collagenesis. However, for indicating the biomaterial in the repair of difficult healing wounds, it is important to have continuity of studies using scaffolds and herbal medicines for better safety in therapy. The molecular analysis also should be performed in future researches to better understand the findings so far, and its absence is considered a limitation in this study.
